# Functional Analysis of the rs774872314, rs116171003, rs200231898 and rs201107751 Polymorphisms in the Human *RORγT* Gene Promoter Region

**DOI:** 10.3390/genes8040126

**Published:** 2017-04-21

**Authors:** Marcin Ratajewski, Marcin Słomka, Kaja Karaś, Marta Sobalska-Kwapis, Małgorzata Korycka-Machała, Anna Sałkowska, Jarosław Dziadek, Dominik Strapagiel, Jarosław Dastych

**Affiliations:** 1 Laboratory of TranscriptionalRegulation, Institute of MedicalBiology, PolishAcademy of Sciences, 93-232 Lodz, Poland; kaja.karas@gmail.com (K.K.); annaswiderek1@wp.pl (A.S.); 2 BiobankLab, Department of MolecularBiophysics, Faculty of Biology and Environmental Protection, University of Lodz, 90-237 Lodz, Poland; m.slomka@biol.uni.lodz.pl (M.S.); sobalska@biol.uni.lodz.pl (M.S.-K.); dominik.strapagiel@biol.uni.lodz.pl (D.S.); 3 MycobacteriumGenetics and Physiology Unit, Institute of MedicalBiology, PolishAcademy of Sciences, 93-232 Lodz, Poland; mkorycka@cbm.pan.pl (M.K.-M.); jdziadek@cbm.pan.pl (J.Dz.); 4 Laboratory of Cellular Immunology, Institute of MedicalBiology, PolishAcademy of Sciences, 93-232 Lodz, Poland; jdastych@cbm.pan.pl

**Keywords:** RORγT, *RORC*, Th17, promoter, polymorphism

## Abstract

RAR-related orphan receptor gamma RORγT, a tissue-specific isoform of the *RORC* gene, plays a critical role in the development of naive CD4+ cells into fully differentiated Th17 lymphocytes. Th17 lymphocytes are part of the host defense against numerous pathogens and are also involved in the pathogenesis of inflammatory diseases, including autoimmune disorders. In this study, we functionally examined four naturally occurring polymorphisms located within one of the previously identified GC-boxes in the promoter region of the gene. The single nucleotide polymorphisms (SNPs) rs774872314, rs116171003 and rs201107751 negatively influenced the activity of the *RORγT* promoter in a gene reporter system and eliminated or reduced Sp1 and Sp2 transcription factor binding, as evidenced by the electrophoretic mobility shift assay (EMSA) technique. Furthermore, we investigated the frequency of these SNPs in the Polish population and observed the presence of rs116171003 at a frequency of 3.42%. Thus, our results suggest that polymorphisms within the *RORγT* promoter occurring at significant rates in populations affect promoter activity. This might have phenotypic effects in immune systems, which is potentially significant for implicating pathogenetic mechanisms under certain pathological conditions, such as autoimmune diseases and/or primary immunodeficiencies (e.g., immunoglobulin E (IgE) syndrome).

## 1. Introduction

RORγ and RORγT proteins are DNA-binding transcription factors transcribed from the same *RORC* gene by a selection of alternative promoters [1,2,3]. Both isoforms are members of the NR1 subfamily of nuclear receptors and, due to their different tissue distributions, they probably play distinct roles in humans [4]. RORγT is a signature transcription factor for Th17 cells and is supposedly directly involved in the regulation of *IL17A* and *IL17F*. Th17 cells protect against numerous pathogens (e.g., *Bacillus anthracis* [5], *Staphylococcus aureus* [6], and *Candida albicans*) [7]; however, clinical observation of increased numbers of Th17 cells in sites of chronic tissue inflammation in patients suffering from multiple sclerosis [8], rheumatoid arthritis [9], psoriasis [10], or Grave’s disease [11] suggest that these lymphocytes are also destructive cells that induce the process of tissue damage in several autoimmune diseases. 

*RORγT* expression is strictly limited to the subset of activated CD4+ cells, while *RORγ* is more broadly expressed. Processes that are responsible for the observed pattern of expression include epigenetic DNA modifications [12] and direct interactions of transcription factors with cis-elements within the 5′-flanking region of the gene. Previously, we cloned the human promoter region of *RORγT* and identified several elements important for its expression, including four E-boxes capable of binding upstream stimulatory factor(USF) transcription factors [3] and two GC-boxes that are able to interact with Sp2 and, to a lesser extent, Sp1 [13]. Both identified GC-boxes seem to be crucial for the activity of the promoter and the expression of the gene in human lymphocytes [13]. After analyzing the National Center for Biotechnology Information (NCBI) Variation Resources [14], we found that four single nucleotide polymorphisms (SNPs) (rs774872314, rs116171003, rs200231898, rs201107751) are located within GC-box 2 of the *RORγT* gene. This prompted us to investigate their functional relevance in human lymphocytes using a luciferase reporter gene assay and an electrophoresis mobility shift assay, and to confirm the distribution of the identified polymorphisms in the Polish population. 

## 2. Materials and Methods

### 2.1. Cell Culture 

A Jurkat (human T cell lymphoblast-like) cell line was purchased from ATCC (Manassas, VA, USA) and maintained under standard conditions in Roswell Park Memorial Institute (RPMI)-1640 (Gibco, ThermoFisher Scientific, Waltham, MA, USA) containing 10% fetal bovine serum (PAN-Biotech GmbH, Aidenbach, Germany) at 37 °C in an atmosphere of 5% CO_2_. 

### 2.2. RORγT Promoter Constructs and Transfection 

All promoter constructs for the *RORγT* gene were described in our previous works [3,13], with the exception of the newly constructed phRORγTp1(−180/+78)Luc, phRORγTp2(−180/+78)Luc, phRORγTp3(−180/+78)Luc, and phRORγTp4(−180/+78)Luc, which were transfected into Jurkat T cells with FuGENE HD (Roche, Basel, Switzerland). The luciferase activity in the cells was measured in 96-well white plates on Infinite^®^ 200 PRO (Tecan, Männedorf, Switzerland). The culture medium was transferred to 96-well transparent plates, for secreted embryonic alkaline phosphatase (SEAP) activity measurements, and lysis buffer was added to the cells before they were frozen at −70°C. After thawing, the luciferase activity was measured. Alkaline phosphatase control activity was determined spectrophotometrically at 405 nm and was used as a transfection efficiency control (The vector pCMV-SEAP was a kind gift from Dr. S. Schlatter, Eidgenoessische Technische Hochschule, Zurich, Switzerland). Luciferase values were normalized per corresponding SEAP activity.

### 2.3. Site-Directed Mutagenesis 

Mutagenesis was performed directly on the pUC18 plasmid carrying −180/+78 sequence of the *RORγT* promoter insert using the polymerase chain reaction (PCR)-based method. Following the reaction, the restriction enzyme DpnI (Fermentas, ThermoFisher Scientific, Waltham, MA, USA) was added to remove the plasmid template. All mutations were identified by restriction enzyme analysis and further verified by sequencing. The mutants were then cloned into the pGL3-Basic vector using the restriction enzymes Acc65I and HindIII (Fermentas, ThermoFisher Scientific). The following primer pairs were used for mutagenesis: 5′-TGGGGCCACCTGGGAGCGGGGGAGCCTGGACCCT-3′ (p1f) and 5′-AGGGTCCAGGCTCCCCCGCTCCCAGGTGGCCCCA-3′ (p1r) (both for the rs774872314 SNP); 5′-TGGGGCCACCTGGGGTCGGGGGAGCCTGGACCCT-3′ (p2f) and 5′-AGGGTCCAGGCTCCCCCGACCCCAGGTGGCCCCA-3′ (p2r) (both for the rs116171003 SNP); 5′-TGGGGCCACCTGGGGGTGGGGGAGCCTGGACCCT-3′ (p3f) and 5′-AGGGTCCAGGCTCCCCCACCCCCAGGTGGCCCCA-3′ (p3r) (both for the rs200231898 SNP); and 5′-TGGGGCCACCTGGGGGCAGGGGAGCCTGGACCCT-3′ (p4f) and 5′-AGGGTCCAGGCTCCCCTGCCCCCAGGTGGCCCCA-3′ (p4r) (both for the rs201107751 SNP).

### 2.4. Electrophoretic Mobility Shift Assays 

An electrophoretic mobility shift assay (EMSA) was performed using infrared dye-labeled (IRD-labeled) DNA probes. Nuclear extracts were prepared from Jurkat cells using a Nuclear Extract Kit (Active Motif, Carlsbad, CA, USA). The following DNA probes were used:

5′-TGGGGCCACCTGGGGGCGGGGGAGCCTGGACCCT-3′ (wild type forward, (wtf)) and 5′-AGGGTCCAGGCTCCCCCGCCCCCAGGTGGCCCCA-3′ (wild type reverse, (wtr)) (both for the wild type sequence) 5′-TGGGGCCACCTGGGAGCGGGGGAGCCTGGACCCT-3′ (p1f) and 5′-AGGGTCCAGGCTCCCCCGCTCCCAGGTGGCCCCA-3′ (p1r) (both for the rs774872314 SNP); 5′-TGGGGCCACCTGGGGTCGGGGGAGCCTGGACCCT-3′ (p2f) and 5′-AGGGTCCAGGCTCCCCCGACCCCAGGTGGCCCCA-3′ (p2r) (both for the rs116171003 SNP); 5′-TGGGGCCACCTGGGGGTGGGGGAGCCTGGACCCT-3′ (p3f) and 5′-AGGGTCCAGGCTCCCCCACCCCCAGGTGGCCCCA-3′ (p3r) (both for the rs200231898 SNP); and 5′-TGGGGCCACCTGGGGGCAGGGGAGCCTGGACCCT-3′ (p4f) and 5′-AGGGTCCAGGCTCCCCTGCCCCCAGGTGGCCCCA-3′ (p4r) (both for the rs201107751 SNP). The DNA probes were incubated on ice with 2.5 µg of nuclear extract in binding buffer containing 10 mM Tris–HCl (pH = 8.0), 50 mMKCl, 18.5 mMNaCl 1 mM Dithiothreitol (DTT), 0.1% IGEPAL, 5% glycerol, and 100 ng of salmon testis DNA (to prevent the nonspecific binding of proteins to the probes). For the competition assay, 50- and 200-fold molar excesses of unlabeled oligonucleotides (wt) were added to the reaction mixture. The DNA-protein complexes were then fractionated on 5% non-denaturing polyacrylamide gels and analyzed on an Odyssey (LiCor Biosciences, Lincoln, NE, USA) infrared fluorescence scanner.

### 2.5. Material and Sample Preparation

The participants were recruited from 2010–2012 for the TESTOPLEK research project and registered as a POPULOUS collection in the BioBank Lab of The Department of Molecular Biophysics of the University of Lodz [15]. Each subject gave written informed consent and completed a questionnaire. The saliva from each individual was collected in Oragene OG-500 DNA collection/storage receptacles (DNA Genotek, Kanata, ON, Canada). This study was approved by the University of Lodz’s Review Board (Ethical approval code: 8/KBBN-UŁ/II/2014). All procedures were performed in accordance with the Declaration of Helsinki (ethical principles for medical research involving human subjects). A total of 5130 participants who declared themselves healthy were involved in the creation of a study group.

The saliva samples were stored at room temperature until initial processing. DNA was manually isolated from 500 µL of saliva according to the manufacturer’s procedure (Prepit L2P, PD-PR-052, DNA Genotek). The elution volume was 50 µL. DNA was quantified using the broad-range Quant-iT™ dsDNA Broad Range Assay Kit (Invitrogen™, Carlsbad, CA, USA). All DNA samples underwent quality control testing using PCR to determine sex, utilizing melting profile analysis and obtaining specific fragments of DNA from samples of human biological material (Patent number PL406569-A1) [16]. Afterwards, the DNA samples included in the study were diluted to 0.2 ng/µL in sterile DNase-free water. All laboratory methods related to sample management were performed according to the standard operating procedures (SOPs) of the BioBank Lab.

### 2.6. High Resolution Melt (HRM) Conditions and Analysis

The single standard reaction mixture (10 µL) was prepared using the Janus^®^ Automated Workstation (Perkin Elmer Inc., Waltham, MA, USA). The mixture was composed of 2×GoTaq^®^ Colorless Master Mix (Promega, Madison, WI, USA), 10×LC Green Plus^®^ dye (BioFire Defense Inc., Salt Lake City, UT, USA), 0.5 µL of 10 µM primers (f, 5′-GTGAATGGGGCCACCTG-3′; r, 5′-GACGACAGGGTCCAGGCT-3′), and 3 µL of DNA (200 pg/µL), and filled with 0.5 µL of water to the final volume. The reaction was performed on a 384-well microplate using the CFX384™ real-time PCR system (Bio-Rad Laboratories Inc., Hercules, CA, USA) (all samples were duplicated). The reaction conditions were as follows: initial denaturation at 95 °C for 3 min, 50 amplification cycles of denaturation at 95 °C for 20 s, and annealing at 60 °C for 30 s. The plate was read after each cycle. Directly afterwards, the melting curve was determined by incubating the plate at 90 °C for 60 s and 40 °C for 60 s, and increasing from 65 °C to 95 °C (in 0.2 °C increments) for 10 s while reading the plate. The obtained data were analyzed with the Bio-Rad Precision Melt Analysis Software, version 1.2 (Bio-Rad Laboratories Inc.).

### 2.7. Detection of Genetic Variation

Genetic variation observed in HRM melting curve analysis was verified by a direct sequencing method for at least three samples representing each cluster. Preparation of samples for sequencing was conducted using specific primers (f, 5′-CTCGGGGGTAGGAGGAGTAG-3′; r, 5′-CCATCTCCCAACAGATCTTGA-3′) according to the previously described protocol [17]. Analysis of sequencing results was performed by CodonCode Aligner software (CodonCode Corporation, Centerville, MA, USA) based on NG_029118 reference sequences (GenBank) [18]. Sequencing results of selected samples were compared with respective clusters of HRM melting curves, and genetic variation was verified.

For the detected polymorphisms, the parameters obtained from GenBank were assigned dbSNP IDs (rs numbers) and coding DNA nucleotide positions, followed by reference sequence NM_001001523.1. These data were used below for the variant nomenclature.

### 2.8. Computational Analysis and Statistics 

Analysis of the *RORγT* gene promoter for potential transcriptional factor binding sites was performed using MatInspector software [19]. Single nucleotide polymorphisms were found in NCBI Variation Resources [14]. Statistical analysis was performed using one-way analysis of variance (ANOVA), followed by Tukey’s post hoc test. A *p*-value of 0.05 or lower was considered statistically significant.

## 3. Results

### 3.1. Effect of the rs774872314, rs116171003, rs200231898 and rs201107751 SNPs on the Activity of the RORγT Promoter

**Figure 1:**
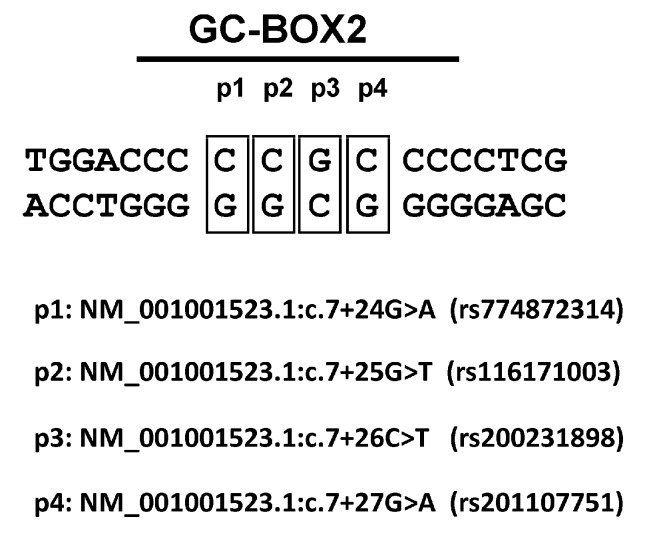
GC-Box 2 within the RAR-related orphan receptor gamma *RORγT* promoter identified using MatInpector Software [19]. Single nucleotide polymorphisms were found in National Center for Biotechnology Information (NCBI) Variation Resources [14].

**Figure 2:**
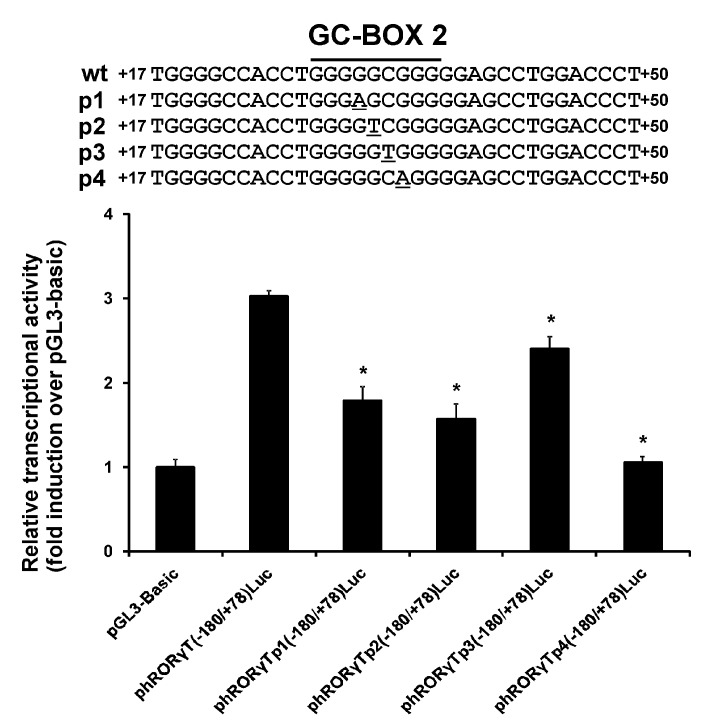
Effect of introduced polymorphisms of GC-box2 on the activity of the *RORγT* proximal promoter in Jurkat cells; phRORγT(−180/+78)Luc contains the wild-type sequence (wt), whereas phRORγTp1(−180/+78)Luc (rs774872314), phRORγTp2(−180/+78)Luc (rs116171003), phRORγTp3(−180/+78)Luc (rs200231898), and phRORγTp4(−180/+78)Luc (rs201107751) contain the mutated sequence. The results shown are from two independent transfections, each performed in triplicate. *Significantly different from the wild type vector (*p* < 0.001; *n* = 6).

First, we checked to determine whether the polymorphisms identified in NCBI Variation Resources containing results of the ExAC project [20], 1000 Genomes project [21,22], and Exome Variant Server [23], were associated with functional differences in related *RORγT* promoter variants in human lymphocytes. Because the rs774872314, rs116171003, rs200231898, and rs201107751 polymorphisms are all located within one of the identified GC-boxes (GC-box 2) (see Figure 1) that is essential for promoter activity, we performed a series of luciferase reporter-based promoter activity assays. We determined the basal activity of the wild-type vector carrying the −180/+78 sequence of the 5′-flanking regions of the *RORγT* gene and mutated vectors. As seen in Figure 2, the wild-type reporter construct is ca. three-fold more active than the promoterless pGL3-Basic vector, whose activity was assigned as 1.0. The rs200231898 polymorphism (p3) decreased the transcriptional activity of the −180/+78 region by only ca. 20%, while the introduction of rs774872314 (p1) and rs116171003 (p2) SNPs resulted in the decreased activity of the *RORγT* promoter by 41% and 48%, respectively. Interestingly, SNP rs201107751 (p4) led to an almost complete loss of activity of the *RORγT* promoter (see Figure 2).

### 3.2. Protein Binding to the Sequences Corresponding to the rs774872314, rs116171003, rs200231898 and rs201107751 Polymorphisms

**Figure 3:**
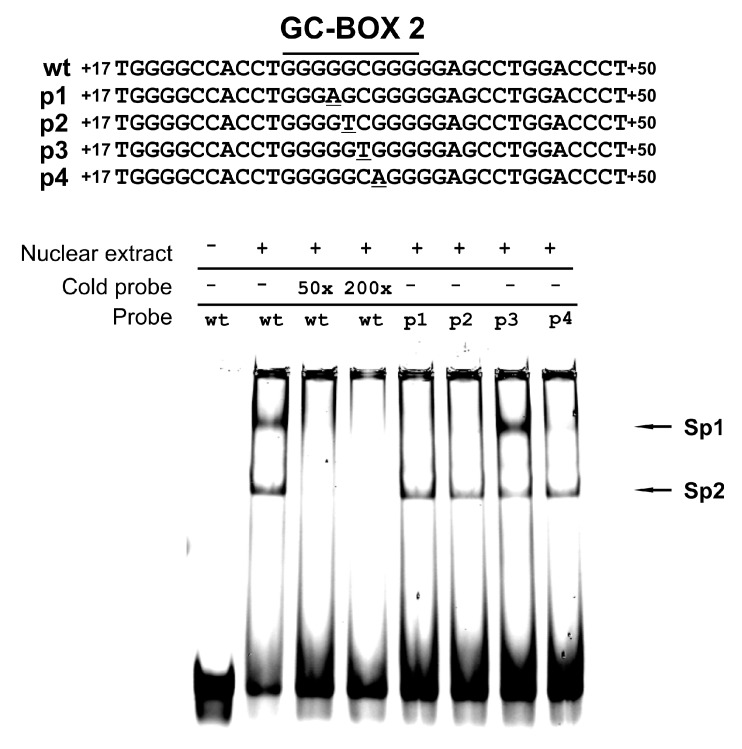
Effect of polymorphisms of GC-box2 on Sp protein binding demonstrated by the electrophoretic mobility shift assay (EMSA).

Effects on factor binding from base exchange in the *RORγT* promoter were determined using EMSA. Based on results from the reporter gene assay, we hypothesized that SNPs could affect protein binding to GC-box 2. To address this hypothesis, we conducted EMSA with probes containing wild-type GC-box 2 and polymorphic residues. The specificity of the observed bands was confirmed using the competition assay with excess unlabeled wild-type probe (see Figure 3). We detected two bands that were previously identified to be occupied by Sp1 (upper) and Sp2 proteins (lower) [13]. We observed elimination (Sp1) and reduction (Sp2) of binding to the p1, p2 and p4 probes. Interestingly, probe p3 gave similar results as the wild-type probe, which confirmed data obtained using the luciferase assay (see Figure 3). 

### 3.3. Analysis of the rs774872314, rs116171003, rs200231898 and rs201107751 Polymorphisms in a Polish Population

**Figure 4:**
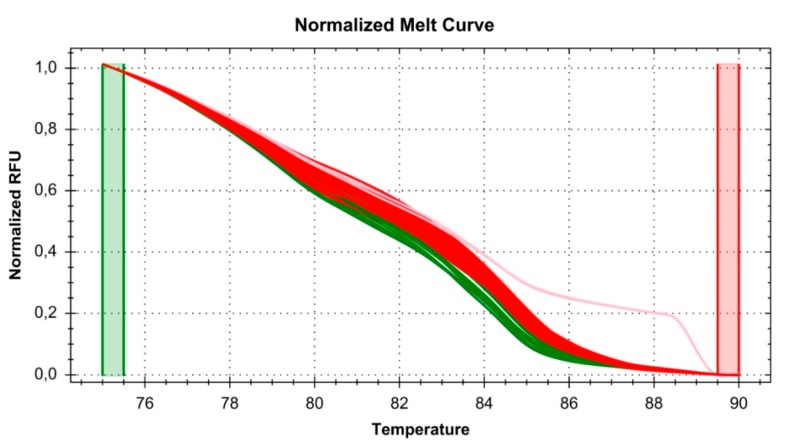
Example of the High Resolution Melt (HRM) assay from scanning 95 DNA samples, run in duplicate. The red cluster is for the reference sequence; the green cluster is for the heterozygous variant of the rs116171003 polymorphism; the pink cluster is for the homozygous variant of the rs116171003 polymorphism. RFU, relative fluorescence units.

Next, we investigated how frequently the polymorphisms identified in the NCBI Variation Resources are found in the Polish population. A total of 5130 human DNA samples were analyzed using HRM methodology (see Figure 4). Of these, 5051 scans were successful, while 79 sample results were inconsistent (no HRM clustering and lack of sequencing results). Among the analyzed samples, we found the rs116171003 polymorphism to have the following frequency of alleles: G = 98.27% and T = 1.73% (Table 1).

As can been seen in Table 1, 4878 donors carried genotype GG, 171 donors were heterozygous carriers, and two individuals were identified as homozygous for the T allele. The rs200231898, rs201107751 and rs774872314 polymorphisms were not detected. Additionally, we found the rs111882199 polymorphism to have the following frequency of alleles: C = 99.02% and G = 0.98% (see Table 1). Because this SNP is not located within the GC-box 2 sequence, it was not investigated further.

## 4. Discussion

Previously, we showed that the 5′-flanking regions of the *RORγT* gene contain two GC-boxes: GC-box 1 (−91/−75 counting from ATG) and GC-box 2 (+28/+36), which are crucial for the activity of the promoter. Furthermore, site-directed mutagenesis suggested that promoter function is dependent on the cooperative interactions of these two boxes [13]. Analyzing public resources for single nucleotide polymorphism data, we found that several naturally occurring polymorphisms are located within one of the GC-boxes (GC-box 2). In the present study, we verified the impact of promoter polymorphisms (see Figure 1) on the activity of the *RORγT* promoter in a reporter system.

The promoter construct carrying the rs200231898 SNP showed only slight differences from the wild-type promoter, but we saw a significant negative effect of the rs774872314, rs116171003, and rs201107751 SNPs on the basal promoter activity (by 41%, 48% and 75%, respectively) (see Figure 2). EMSA performed with mutated and wild-type probes confirmed results obtained in the gene reporter system. We observed a significant decrease in protein binding to the rs774872314, rs116171003, and rs201107751 probes, while the probe carrying the rs200231898 polymorphism was nearly unaffected (see Figure 3). This is in agreement with previous studies showing that having G-residues at positions one, two and four in the GGCGG core sequence is critical for the formation of DNA-Sp protein complexes, while a C mutation at position three has little or no effect [24]. It should be noted that the substitution of polymorphic bases in oligonucleotide probes resulted in a significant decrease in Sp1 binding, while the binding of Sp2 was affected to a much lesser degree (see Figure 3). These observations are of interest to better understand the molecular mechanism governing Sp1- and Sp2-dependent *RORγT* promoter activity. Thus, polymorphisms demonstrating the strongest inhibitory effect on promoter activity (see Figure 2) have also demonstrated the strongest detrimental effect on Sp1 binding (see Figure 3), which is consistent with the hypothesis that Sp1 is directly involved in *RORγT* promoter activity. This is somehow contradictory to our previous observation that depletion of Sp1, in contrast to depletion of Sp2, did not abolish *RORγT* expression in lymphocytes [13]. On the other hand, we detected promoter occupancy by Sp1 using a chromatin immunoprecipitation assay. While analyzing these observations, it should be acknowledged that Sp1 is known as a stronger transactivator compared to Sp2 [25], and that siRNA experiments were performed in cells in which Sp1 is more highly expressed than Sp2. These two factors might diminish the effects that significantly reduced amounts of available Sp1 have on expression of *RORγT*. Taken together, the most likely explanation for these observations is the hypothesis that the transcriptional regulation of *RORγT*, coordinated with the action of Sp1 and Sp2, possibly involves the direct interaction of these two proteins and/or their interaction with other proteins. In this regard, Sp1 in other systems has already been shown to interact with several proteins [26,27,28,29,30].

RORγT is considered to be a signature transcription factor for Th17 lymphocytes, as its ectopic expression in CD4+ cells is sufficient to develop the Th17-like phenotype [31]. RORγT is also able to upregulate the expression of Th17-specific interleukins *IL17A* and *IL17F* by binding to ROR-response elements (ROREs) present in their promoters [32,33]. Th17-derived IL17A and IL17F are linked to the pathogenesis of some autoimmune diseases, including multiple sclerosis [8], rheumatoid arthritis [9], psoriasis [10], and Grave’s disease [11]. However, both interleukins are also essential for host defense against pathogens, such as *Bacillus anthracis* [5], *Staphylococcus aureus* [6], and *Candida albicans* [7,34]. Genome screening studies allowed for the identification of many new polymorphisms in both coding and non-coding sequences. Most of them do not have physiological manifestations, but some affect gene functions or expression [35,36,37]. In this context, the presence of rs201107751, the SNP that nearly reduced promoter activity levels to those observed for the promoterless pGL3-Basic vector, might also significantly reduce *RORγT* expression. Very low expression of *RORγT* is observed in carriers of mutations in the *STAT3* gene. This deficiency of *RORγT* results in the lack of Th17 cells in the bloodstream [38,39] and the development of primary immunodeficiency characteristics, called IgE syndrome (Job’s syndrome), by recurring pneumonia and mucocutaneous candidiasis caused by *Staphylococcus aureus* and *Candidia albicans*. Other SNPs, namely, rs774872314 and rs116171003, also have significant potential to impair the expression of *RORγT*, which leads to changes in the phenotype of immune cells. Thus, the rs774872314 and rs116171003 SNPs can reduce the activity of this promoter and, in turn, decrease the expression of *RORγT*. This might be considered a potential protective factor that decreases the chances for the development of autoimmunological disorders that depend on Th17 cells. This would be similar to the rs3811046 and rs3811047/rs2723186 polymorphisms in the IL37 gene [40]. This is of special interest, as one of these SNPs, namely, rs116171003, (Table 1) occurs in the investigated Polish population with significant (3.42%) frequency. This creates opportunity for further experimental exploration of the hypothesis that links this SNP with the likelihood of certain immunological disorders in this population. Because the distribution of alleles occurs at different frequencies in different populations [41,42], a similar hypothesis for other SNPs might also be verified in the future.

In conclusion, our results clearly show that naturally occurring genetic polymorphisms in Sp-binding motifs of the *RORγT* promoter are associated with significant differences in the functionality of this promoter. These observations justify further studies to elucidate the potential effects of the rs774872314, rs116171003, and rs201107751 polymorphisms on the susceptibility of their carriers to certain immunological disorders.

## Figures and Tables

**Table 1:** Summary of *RORγT* promoter variants detected by HRM scanning.

SNP	Genotype	N	%	MAF
NM_001001523.1:c.7+25G>T (rs116171003)	GG	4878	96.575	(T) 0.017
	GT	171	3.385	
	TT	2	0.040	
NM_001001523.1:c.7+34C>G (rs111882199)	CC	4993	98.852	(G) 0.006
	CG	57	1.128	
	GG	1	0.020	

SNP, single nucleotide polymorphisms; N, number of samples; MAF, Minor allele frequency.

## References

[1] Medvedev A., Chistokhina A., Hirose T., Jetten A.M. (1997). Genomic structure and chromosomal mapping of the nuclear orphan receptor RORγ (RORC) gene. Genomics.

[2] Villey I., de Chasseval R., de Villartay J.P. (1999). RORγT, a thymus-specific isoform of the orphan nuclear receptor RORγ/TOR, is up-regulated by signaling through the pre-T cell receptor and binds to the tea promoter. Eur. J. Immunol..

[3] Ratajewski M., Walczak-Drzewiecka A., Salkowska A., Dastych J. (2012). Upstream stimulating factors regulate the expression of RORγT in human lymphocytes. J. Immunol..

[4] Jetten A.M. (2009). Retinoid-related orphan receptors (RORs): Critical roles in development, immunity, circadian rhythm, and cellular metabolism. Nucl. Recept. Signal..

[5] Harris K.M., Ramachandran G., Basu S., Rollins S., Mann D., Cross A.S. (2014). The IL-23/Th17 axis is involved in the adaptive immune response to *Bacillus anthracis* in humans. Eur. J. Immunol..

[6] Lin L., Ibrahim A.S., Xu X., Farber J.M., Avanesian V., Baquir B., Fu Y., French S.W., Edwards J.E., Spellberg B. (2009). Th1-Th17 cells mediate protective adaptive immunity against *Staphylococcus aureus* and *Candida albicans* infection in mice. PLoSPathog..

[7] Huang W., Na L., Fidel P.L., Schwarzenberger P. (2004). Requirement of interleukin-17a for systemic anti-*Candida albicans* host defense in mice. J. Infect. Dis..

[8] Kebir H., Kreymborg K., Ifergan I., Dodelet-Devillers A., Cayrol R., Bernard M., Giuliani F., Arbour N., Becher B., Prat A. (2007). Human Th17 lymphocytes promote blood-brain barrier disruption and central nervous system inflammation. Nat. Med..

[9] Hirota K., Hashimoto M., Yoshitomi H., Tanaka S., Nomura T., Yamaguchi T., Iwakura Y., Sakaguchi N., Sakaguchi S. (2007). T cell self-reactivity forms a cytokine milieu for spontaneous development of IL-17^+^Th cells that cause autoimmune arthritis. J. Exp. Med..

[10] Li J., Chen X., Liu Z., Yue Q., Liu H. (2007). Expression of Th17 cytokines in skin lesions of patients with psoriasis. J. HuazhongUniv. Sci. Technol. Med. Sci..

[11] Zheng L., Ye P., Liu C. (2013). The role of the IL-23/IL-17 axis in the pathogenesis of graves’ disease. Endocr. J..

[12] Schmidl C., Hansmann L., Andreesen R., Edinger M., Hoffmann P., Rehli M. (2011). Epigenetic reprogramming of the RORC locus during in vitro expansion is a distinctive feature of human memory but not naive Treg. Eur. J. Immunol..

[13] Ratajewski M., Walczak-Drzewiecka A., Gorzkiewicz M., Salkowska A., Dastych J. (2016). Expression of human gene coding RORγT receptor depends on the Sp2 transcription factor. J. Leukoc. Biol..

[14] NCBI Resource Coordinators (2015). Database resources of the National Center for Biotechnology Information. Nucleic Acids Res..

[15] Strapagiel D., Sobalska-Kwapis M., Slomka M., Marciniak B. (2016). Biobank Lodz—DNA based biobank at the University of Lodz, Poland. Open J. Bioresour..

[16] Strapagiel D., Majewska M., Słomka M., Janik K., Sobalska M., Bartosz G. (2016). Method for Determining sex, Involves Utilizing Melting Profile Analysis Technique, and Obtaining Specific Fragments of DNA by PCR-Based DNA from Samples of Human Biological Material.

[17] Slomka M., Sobalska-Kwapis M., Korycka-Machala M., Bartosz G., Dziadek J., Strapagiel D. (2015). Genetic variation of the ABC transporter gene ABCC1 (multidrug resistance protein 1-MRP1) in the Polish population. BMC Genet..

[18] Benson D.A., Cavanaugh M., Clark K., Karsch-Mizrachi I., Lipman D.J., Ostell J., Sayers E.W. (2013). GenBank. Nucleic Acids Res..

[19] Cartharius K., Frech K., Grote K., Klocke B., Haltmeier M., Klingenhoff A., Frisch M., Bayerlein M., Werner T. (2005). Matinspector and beyond: Promoter analysis based on transcription factor binding sites. Bioinformatics.

[20] Lek M., Karczewski K.J., Minikel E.V., Samocha K.E., Banks E., Fennell T., O’Donnell-Luria A.H., Ware J.S., Hill A.J., Cummings B.B. (2016). Analysis of protein-coding genetic variation in 60,706 humans. Nature.

[21] Auton A., Brooks L.D., Durbin R.M., Garrison E.P., Kang H.M., Korbel J.O., Marchini J.L., McCarthy S., McVean G.A., Abecasis G.R. (2015). A global reference for human genetic variation. Nature.

[22] Sudmant P.H., Rausch T., Gardner E.J., Handsaker R.E., Abyzov A., Huddleston J., Zhang Y., Ye K., Jun G., Hsi-Yang Fritz M. (2015). An integrated map of structural variation in 2,504 human genomes. Nature.

[23] Exome Variant Server, NHLBI GO Exome Sequencing Project (ESP), Seattle, WA, USA. http://evs.gs.washington.edu/EVS/.

[24] Hoppe K.L., Francone O.L. (1998). Binding and functional effects of transcription factors Sp1 and Sp3 on the proximal human lecithin: Cholesterol acyltransferase promoter. J. Lipid Res..

[25] Moorefield K.S., Fry S.J., Horowitz J.M. (2004). Sp2 DNA binding activity and trans-activation are negatively regulated in mammalian cells. J. Biol. Chem..

[26] Courey A.J., Holtzman D.A., Jackson S.P., Tjian R. (1989). Synergistic activation by the glutamine-rich domains of human transcription factor Sp1. Cell.

[27] Pascal E., Tjian R. (1991). Different activation domains of Sp1 govern formation of multimers and mediate transcriptional synergism. Genes Dev..

[28] Emili A., Greenblatt J., Ingles C.J. (1994). Species-specific interaction of the glutamine-rich activation domains of Sp1 with the TATA box-binding protein. Mol. Cell. Biol..

[29] Torigoe T., Izumi H., Yoshida Y., Ishiguchi H., Okamoto T., Itoh H., Kohno K. (2003). Low pH enhances Sp1 DNA binding activity and interaction with TBP. Nucleic Acids Res..

[30] Hoey T., Weinzierl R.O., Gill G., Chen J.L., Dynlacht B.D., Tjian R. (1993). Molecular cloning and functional analysis of drosophila TAF110 reveal properties expected of coactivators. Cell.

[31] Crome S.Q., Wang A.Y., Kang C.Y., Levings M.K. (2009). The role of retinoic acid-related orphan receptor variant 2 and IL-17 in the development and function of human CD4+ T cells. Eur. J. Immunol..

[32] Zhang F., Meng G., Strober W. (2008). Interactions among the transcription factors Runx1, RORγT and Foxp3 regulate the differentiation of interleukin 17-producing T cells. Nat. Immunol..

[33] Yang X.O., Pappu B.P., Nurieva R., Akimzhanov A., Kang H.S., Chung Y., Ma L., Shah B., Panopoulos A.D., Schluns K.S. (2008). T helper 17 lineage differentiation is programmed by orphan nuclear receptors RORα and RORγ. Immunity.

[34] Onishi R.M., Gaffen S.L. (2010). Interleukin-17 and its target genes: Mechanisms of interleukin-17 function in disease. Immunology.

[35] Yamaguchi-Kabata Y., Shimada M.K., Hayakawa Y., Minoshima S., Chakraborty R., Gojobori T., Imanishi T. (2008). Distribution and effects of nonsense polymorphisms in human genes. PLoS ONE.

[36] Dunna N.R., Vuree S., Kagita S., Surekha D., Digumarti R., Rajappa S., Satti V. (2012). Association of GSTP1 gene (I105V) polymorphism with acute Leukaemia. J. Genet..

[37] Pastinen T., Ge B., Hudson T.J. (2006). Influence of human genome polymorphism on gene expression. Hum. Mol. Genet..

[38] Ma C.S., Chew G.Y., Simpson N., Priyadarshi A., Wong M., Grimbacher B., Fulcher D.A., Tangye S.G., Cook M.C. (2008). Deficiency of Th17 cells in hyper IgE syndrome due to mutations in stat3. J. Exp. Med..

[39] Eyerich K., Foerster S., Rombold S., Seidl H.P., Behrendt H., Hofmann H., Ring J., Traidl-Hoffmann C. (2008). Patients with chronic mucocutaneous candidiasis exhibit reduced production of Th17-associated cytokines IL-17 and IL-22. J. Investig. Dermatol..

[40] Yan N., Meng S., Song R.H., Qin Q., Wang X., Yao Q., Jiang Y., Jiang W., Shi L., Xu J. (2015). Polymorphism of IL37 gene as a protective factor for autoimmune thyroid disease. J. Mol. Endocrinol..

[41] Wang Z., Wang B., Tang K., Lee E.J., Chong S.S., Lee C.G. (2005). A functional polymorphism within the MRP1 gene locus identified through its genomic signature of positive selection. Hum. Mol. Genet..

[42] Umamaheswaran G., Kumar D.K., Adithan C. (2014). Distribution of genetic polymorphisms of genes encoding drug metabolizing enzymes & drug transporters—A review with Indian perspective. Indian J. Med. Res..

